# Precision Public Health Initiatives in Cancer: Proceedings from the Transdisciplinary Conference for Future Leaders in Precision Public Health

**DOI:** 10.1186/s12919-022-00234-x

**Published:** 2022-07-07

**Authors:** Caitlin G. Allen, Erin Turbitt, Amelia K. Smit, Lauren E. Passero, Dana Lee Olstad, Ashley Hatch, Latrice Landry, Megan C. Roberts

**Affiliations:** 1 Medical University of South Carolina, Charleston, USA; 2 University of Technology Sydney, Sydney, Australia; 3 The Daffodil Centre, The University of Sydney, a joint venture with Cancer Council NSW, Sydney, Australia; 4 Melanoma Institute Australia, The University of Sydney, Sydney, Australia; 5 The University of Sydney, Faculty of Medicine and Health, Sydney School of Public Health, Sydney, Australia; 6 University of North Carolina, Chapel Hill, USA; 7 University of Calgary, Calgary, Canada; 8 Harvard University, Cambridge, USA

**Keywords:** Precision public health, Transdisciplinary, Convening

## Abstract

**Background:**

Precision public health is an emergent field that requires transdisciplinary collaborations and leverages innovative approaches to improve population health. These opportunities have inspired a new generation of precision public health researchers. Despite burgeoning interest in precision public health, there are limited opportunities for researchers to convene and continue the momentum of this field.

**Methods:**

The *Transdisciplinary Conference for Future Leaders in Precision Public Health* was the among the first events to bring together international researchers and practitioners to learn, network, and agenda set for the future of the field. The conference took place virtually on October 14 and 15, 2021.

**Results:**

The conference spanned two days and featured a keynote address, speakers from public health disciplines who are international leaders in precision-based research, networking opportunities, a poster session, and research agenda setting activities.

**Conclusion:**

The conference was a critical first step to creating a shared international conversation about precision public health, especially among early-stage investigators. This allowed attendees to continue building their individual skills and international collaborations to support the growth of the field of precision public health.

## Rationale for the transdisciplinary conference for future leaders in precision public health

Precision public health is an emergent field of research that requires transdisciplinary collaborations, as it integrates public health and precision medicine disciplines to provide “the right *intervention* to the right *population* at the right time” [1]. Precision-based approaches to public health can leverage genomic discoveries to improve population health, inspiring a new generation of precision public health researchers at the forefront of the field. Wide-ranging research opportunities have been generated to improve data integration and analysis methods, health policies, health equity, and translation of clinical genomic models into population screening [2–4]. Despite significant interest in precision public health worldwide, there are limited opportunities for early career researchers (i.e., completed terminal research degree or residence within the past 10 years) to convene to gain the necessary knowledge, skills and networks to engage in this research. To date, there has only been one dedicated conference for precision public health that allows investigators from both public health and precision medicine disciplines to come together: the Precision Public Health Asia conference, last held in Australia in 2018. To overcome these challenges, in October 2021, a group of international early-stage investigators hosted a virtual conference, the *Transdisciplinary Conference for Future Leaders in Precision Public Health*” [5]. This conference was designed to bring together experts in the field and a new generation of precision public health researchers to build capacity, establish research priorities, and offer networking opportunities. This manuscript describes the conference goals and discussions during expert talks, summarizes the meeting, including attendee evaluation of the conference from attendees, and discusses next steps.

## Planning and conference preparation

The* Transdisciplinary Conference for Future Leaders in Precision Public Health* was held as a virtual meeting hosted by the University of North Carolina, Chapel Hill. The international organizing committee consisted of early career investigators who have collaborated on a number of thought pieces and projects related to precision public health since 2018 [3, 4, 6]. We received funding from the National Cancer Institute through the NIH Support for Conferences and Scientific Meetings (Parent R13 Clinical Trial Not Allowed, 1R13CA261073), as well as UNC Eshelman School of Pharmacy, UNC Lineberger Comprehensive Cancer Program, and UNC Program for Precision Medicine in Healthcare in Summer 2021 to begin planning for the October 2021 conference.

Conference planning involved the development of advertising materials, including a dedicated Twitter page (https://twitter.com/PPHFutureLeader) and conference website (https://pharmacy.unc.edu/pharmsciconference/). Advertising occurred through the conference organizer’s personal and professional networks and via email to reach academic, government, and industry audiences. Specifically, we promoted conference registration, sharing the opportunity to participate in the virtual poster session, and sending reminders about sessions. Conference planning included development of advertising materials, a dedicated Twitter page, and conference website. The Twitter page was used to promote conference registration, share the opportunity to participate in the virtual poster session, and send reminders about sessions. Once a participant completed free registration, they were added to an email list to receive the conference Zoom link.

The conference was initially designed to take place in-person; however, we modified the conference to take place virtually due to ongoing concerns about the COVID-19 pandemic and travel restrictions. This required us to shift planned networking activities and poster sessions to a format appropriate for virtual interactions. Due to the international nature of the conference, we were limited in ability to provide networking activities in time zones convenient to the full audience. To overcome this, we used our conference-specific Twitter account and hashtag for the conference to promote discussion between sessions and encouraged additional networking beyond planned conference activities. In addition, we used conference workshop activities (research priority setting) as an opportunity to network.

## The Transdisciplinary Conference for Future Leaders in Precision Public Health conference proceedings

We hosted the virtual conference on October 14 and 15, 2021 The goals of the conference were to: (1) gain advanced content and methods expertise in precision public health, (2) develop research priorities, and (3) establish transdisciplinary networks that will convene regularly.

A total of 112 participants registered for the conference, with 52 unique attendees participating in activities over the course of the conference. Registrants self-reported their public health discipline, which included: health behavior (*n =* 36, 32.14%), epidemiology (*n =* 30, 26.79%), health policy (*n =* 43, 38.4%), biostatistics (*n =* 14, 12.50%), and environmental health (*n =* 9, 8.1%). The majority of participants were from North America (*n =* 45, 86.5%) and Asia Pacific was the next highest represented region (*n =* 5, 9.6%) (Table 1).

**Table 1:** Conference participant information

	All Registrants (*n =* 112)	Conference Attendees (*n =* 52)	Workshop Attendees (*n =* 15)
Geography	*n* (%)	*n* (%)	*n* (%)
Asia Pacific	12 (10.7%)	5 (9.6%)	2 (13.3%)
Europe	1 (0.9%)	0 (0%)	0 (0%)
North America	97 (86.6%)	45 (86.5%)	12 (80.0%)
South America	2 (1.8%)	2 (3.9%)	1 (6.7%)
Public Health Discipline^a^			
Health Behavior	36 (32.1%)	15 (28.9%)	5 (33.3%)
Epidemiology	30 (26.8%)	17 (32.7%)	5 (33.3%)
Health Policy	43 (38.4%)	15 (28.9%)	5 (33.3%)
Biostatistics	14 (12.5%)	7 (13.5%)	2 (13.3%)
Environmental Health	9 (8.0%)	7 (13.5%)	0 (0%)

^a^Public Health Discipline was self-reported by participants and more than one discipline could be selected

The agenda included a Plenary Session, six speakers with expertise in precision public health from key public health disciplines, including: epidemiology, nutrition, environmental health sciences, health services research, social and behavioral sciences, and biomedical informatics. The conference concluded with closing remarks and a summary of next steps.

### Dr. Wylie Burke, The emerging field of precision public health

Dr. Burke provided a keynote address to set the stage for the conference attendees about the field of precision public health. This included defining precision medicine, the utility of genetics as part of precision-based approaches, and two examples of precision public health. Dr. Burke included implications for precision public health, noting the value of new knowledge as tools for the field, while also indicating the importance of standard approaches in epidemiology, population screening, and community-based interventions.

### Dr. Peter Kanetsky, Prioritizing health equity in cancer genetic epidemiology research

Dr. Kanetsky described how precision public health approaches have been infused to his program of research. This included examples from research in melanoma, beginning with the translation of genetic epidemiology findings to clinical and public health practice. Dr. Kanetsky specifically discussed the IMPACT-ME study, [7] the Melanoma Genomics Managing Your Risk Study, [8] and SOMBRA study all focused on intervention research to reduce melanoma and skin cancer [9]. Future directions in cancer genetic epidemiology research include hybrid type 1 effectiveness-implementation studies, examining the long-term impacts of precision prevention approaches, cross-generational effects of precision prevention, and increasing inclusion of Hispanics in longitudinal melanoma research.

### Dr. Dawn Wilson, Gene by environment interaction on weight-related outcomes in underserved african american adults

Dr. Wilson framed her presentation using the multi-level bioecological framework. She described genetic risk and social environment factors that impact obesity-related outcomes in African American adults, specifically, the Path Trial, which was an intervention that addressed neighborhood factors and a genetic risk index on waist circumference. The study illuminated opportunities for prevention that account for multi-level influences of neighborhood and genetic factors, stressing the importance of integrating common disease risk assessments into clinical practice, using a systems approach to incorporate multilevel factors in prevention, and advocating for public policy interventions.

### Dr. Andrea Baccarelli, Gene X environment in the context of environmental health sciences

Dr. Baccarelli described approaches to detecting unhealthy environments through DNA methylation (epigenetics), extracellular RNA, and epitranscriptomics. This presentation focused on unique opportunities to address how disease risk factors (e.g., physical inactivity, environmental risk factors such as air pollution and smoking, diet, and metabolic risk) may be captured by our cells to predict the future risk of disease. Recommended future directions in gene X environment include application of new methods to large cohort studies and multiple phenotypes, as well as opening new paths to understand mechanisms and foster prevention.

### Dr. Alanna Kulchak Rahm, Implementation of precision prevention in oncology

Dr. Kulchak Rahm shared examples of precision prevention using implementation science approaches. She described current efforts to advance Lynch Syndrome screening using universal tumor screening (IMPULSS) and traceback cascade screening to identify individuals with ovarian cancer and use cascade screening to inform at-risk family members (FACTS). Dr. Kulchak Rahm included future opportunities to advance precision public health through the integration of genomics, a learning health system, implementation science, and engagement.

### Dr. Chanita Hughes Halbert, Precision public health to address minority health and health disparities

Dr. Hughes Halbert provided foundational definitions of health equity in precision public health, including approaches to develop and implement lifestyle interventions to individuals who are most likely to benefit and retain these experiences in diverse populations. Dr. Hughes Halbert presented a wide-range of research studies demonstrating the utility of precision approaches in community settings and health systems.

### Dr. Chirag Patel, Estimating the architecture of exposome-phenome association and implications for precision public health

Dr. Patel discussed the influence of the genome (i.e., variants), and exposome (e.g., infectious agents, nutrients, pollutants) on an individual’s phenome (i.e., gene expression). This included sharing ongoing research on exposome-wide association studies and efforts to build poly-exposure risk scores to better characterize factors associated with health outcomes.

### Dr. Muin Khoury, From public health genomics to precision public health: onto the next generation!

Dr. Khoury shared a vision for the next generation of precision public health. This included describing the movement from public health genomics toward precision public health, shifting beyond applications of precision medicine to populations and toward use of precision public health to advance population health, and the impact of precision public health in COVID-19 era. Dr. Khoury integrated examples from conference presentations to summarize and invigorate next phases of precision public health.

### Workshops

We also hosted three workshops to help envision success and prioritize precision public health research topics. The first two-hour workshop titled, “Envisioning Success and Prioritizing PPH Research Topics” lasted two hours and included breakout sessions, arranged according to attendees’ public health discipline (e.g., health behavior, health services research, epidemiology, biostatistics), to discuss potential research priorities for the field. At the conclusion of this session, two members from the conference organizing committee reviewed all possible research priorities, distilled them into ten specific themes, and defined these themes based on discussions from the first workshop. During the second workshop, the conference organizing committee reviewed the key themes with attendees and asked attendees to prioritize them using a brief survey. Finally, during the third workshop, individuals participated in one of three breakout sessions based on the identified research priorities. During this session, the conference participants further refined the research priorities and came up with linked objectives. Results from the workshop are described elsewhere [5].

### Networking and posters

To support networking, we hosted a live Twitter poster session. The organizing committee solicited abstracts from conference participants prior to the conference and used a standard rubric to select a winner of the poster competition. During the Twitter poster session, all authors posted an image of their poster and responded to questions from other attendees about its scientific content.

## Evaluation of Transdisciplinary Conference for Future Leaders in Precision Public Health conference

We provided a REDCap survey to participants at the conclusion of the conference via our conference list serv. Because we plan to offer the conference on an annual basis, our goal for the evaluation was to identify areas of the conference that worked well and areas that could be improved upon in the future.

### Speaker evaluations

We assessed the quality of each speaker rated on a scale of 1 (poor) to 5 (excellent) and calculated mean and standard deviation. Quality of information provided (4.4, 0.6), quality of visual aids (4.4, 0.7), knowledge of subjects (4.7, 0.5), presentation skills (4.6, 0.6), and quality of overall presentation (4.6, 0.5) were highly rated. Participants also rated their level of agreement about how helpful the conference sessions were on a scale of 1 (strongly disagree) to 5 (strongly agree). Participants agreed that the conference advanced their understanding of precision public health (4.5, 0.5), and covered materials that would be useful in their work (4.2, 0.9) Table 2.

**Table 2:** Speaker Evaluations (*n =* 22)

Criteria	Mean	Standard Deviation
**Quality of Information provided**	4.4	0.6
**Quality of Visual Aids**	4.4	0.7
**Presenter’s knowledge of Subjects**	4.7	0.5
**Presenter’s presentation skills**	4.6	0.6
**Quality of overall presentation**	4.6	0.5
Session advanced understanding of precision public health*	4.5	0.6
Session covered materials that would be useful in work*	4.2	0.9

Likert Scale: 1 = Poor, 2 = Fair, 3 = Good, 4 = Very Good, 5 = Excellent, *Likert Scale: 1 = Strongly Disagree, 2 = Disagree, 3 = Neither agree or disagree, 4 = Agree, 5 = Strongly Agree

### Workshop evaluations

We also assessed the quality of the three workshops held during the conference (scale 1 [poor] to 5 [excellent]). The workshops were designed as an opportunity for attendees to discuss priorities for the field of precision public health. The quality of information presented (4.2, 0.45), quality of visual aids and handouts (4, 0.71), facilitator’s knowledge of the subject (3.4, 1.52), facilitator’s ability to guide consensus building activities (3.4, 1.5), and quality of the consensus building activities (3.6, 0.9) were ranked primarily as good to very good. Individuals agreed that the consensus workshop helped to develop research priorities for the field (4.4, 0.6) and covered materials that may be useful in the participant’s own work (4.0, 0.7) (Table 3).

**Table 3:** Consensus Building Workshop Evaluations (*n =* 5)

Criteria	Mean	Standard Deviation
**Quality of information presented**	4.2	0.5
**Quality of visual aids and handouts**	4	0.7
**Facilitator’s knowledge of the subject**	3.4	1.5
**Facilitator’s ability to guide consensus building activities**	3.4	1.5
**Quality of overall Consensus Building Activities**	3.6	0.9
The Consensus Building Workshops helped to develop Research Priorities for the field of Precision Public Health*	4.4	0.6
The Consensus Building Workshops covered materials that will be useful in my own work*	4.0	0.7

Likert Scale: 1 = Poor, 2 = Fair, 3 = Good, 4 = Very Good, 5 = Excellent, *Likert Scale: 1 = Strongly Disagree, 2 = Disagree, 3 = Neither agree or disagree, 4 = Agree, 5 = Strongly Agree

### Overall satisfaction

Finally, we requested that participants share their overall satisfaction with the conference on scale of poor (1) to excellent (5). This included: quality of virtual facilities, quality of speakers, quality of consensus building workshop, quality of poster session, availability of networking opportunities, and the overall meeting experience. This included agreement about the quality of the virtual facilities (4.4, 0.5), quality of speakers (4.9, 0.4), quality of consensus building workshops (4.3, 0.5), quality of poster session (4.0, 1.0), and availability of networking opportunities (3.7, 1.2). These were rated as good to very good, with lower rating of the availability of networking opportunities. We also asked level of agreement (strongly disagree [1] to strongly agree [5]) about whether the conference met its stated objectives (4.3, 0.5), satisfaction with overall conference experience (4.1, 0.7), and whether the conference increased likelihood to engage in precision public health efforts (3.7, 1.4) Table 4.

**Table 4:** Overall Satisfaction (*n =* 7)

Criteria	Mean	Standard Deviation
**Quality of virtual facilities**	4.4	0.5
**Quality of speakers**	4.9	0.4
**Quality of consensus building workshop**	4.3	0.5
**Quality of poster session**	4	1
**Availability of networking opportunities**	3.7	1.2
**The conference met its stated objective to address research, training, and networking opportunities for early career faculty and trainees in precision public health in oncology**	4.3	0.5
**I am satisfied with my overall conference experience**	4.1	0.7
**Attending this conference made me more likely to engage**	3.7	1.4

Likert Scale: 1 = Poor, 2 = Fair, 3 = Good, 4 = Very Good, 5 = Excellent, *Likert Scale: 1 = Strongly Disagree, 2 = Disagree, 3 = Neither agree or disagree, 4 = Agree, 5 = Strongly Agree

## Discussion

This paper describes the planning and evaluation of *Transdisciplinary Conference for Future Leaders in Precision Public Health* that was held in October 2021. The planning committee consisted of a team of international early-stage investigators. Through this conference, we sought to: (1) gain advanced content and methods expertise in precision public health, (2) develop research priorities, and (3) establish transdisciplinary networks that will convene regularly.

Our conference evaluation revealed that attendees were able to increase their knowledge related to precision public health. This was achieved through the presentation of high-quality information, visual aids, and engagement with individuals who are working in precision public health. Specifically, participants found that sessions helped advance their knowledge of the field and covered materials that could be useful in their work. Conference attendees were highly engaged as part of the one-hour presentations, asking questions through the chat feature of the virtual platform, and all sessions were recorded and publicly available to ensure ongoing access to speaker materials.

The organizing committee leveraged our networks to identify and invite individuals across public health disciplines who are leading researchers in precision public health. Providing an introductory keynote session to define terms and review the history of precision public health, as well as a concluding keynote session to summarize the conference themes helped attendees increase their knowledge of precision public health and its application within various fields of research. We decided to provide these sessions “live” to allow for participant engagement in real-time; however, the publicly accessible recordings have allowed for interested individuals to review materials and extended the reach of speakers (e.g., Muin Khoury recording currently has greater than 300 views). One opportunity for future virtual conferences would be to pre-record expert presentations or reduce the length of these talks and host question and answer sessions that allow ample opportunity to discuss the presentation content and maximize interaction between early-stage investigators and leaders in the field.

Another key objective was to develop research priorities. Our three-part consensus building workshop was designed to provide participants with the opportunity to identify research priorities to advance the field. This resulted in three priorities: (1) equity and access, (2) improving tools and metrics for evaluation, and (3) applying principles of implementation research to precision public health [5]. Overall, these sessions were rated lower than speaker sessions in terms of their quality. Our initial conference plans would have engaged individuals in-person through break-out sessions and additional discussions. While we tried to facilitate a similar experience for participants in a virtual setting, conducting break-out think tank style sessions virtually is challenging. For example, we had substantial differences in time zones (up to 14 hours), resulting in difficulty in identifying times that were accessible to all audiences and limited attendance for certain sessions. We used best practice for facilitation of the consensus building workshop (e.g., provided instructions and worksheets) and included an external facilitator; however, attendance across consensus building workshops was low due to the time of day for attendees (e.g., late in the evening for those in North America or early in the morning for those in Australia). One strength of our approach was the use of a survey to request all conference attendee’s feedback about which of the 10 research priorities the group should focus on describing as part of the final consensus building workshop. Efforts are now underway to continue working on the research priorities through two work groups: (1) Implementation and Evaluation, and (2) Health Equity.

Our final aim was to establish a network of early-stage investigators who will convene regularly. We facilitated networking opportunities throughout the conference, including as part of the consensus building workshop and Twitter poster session. The availability of networking opportunities was ranked as 3.7, demonstrating opportunities to improve networking in future conferences. We sought to identify times for networking that were accessible to individuals from time zones across the world. Our original conceptualization of an in-person conference would have allowed for further networking; however, this was a difficult task in the virtual setting. Additionally, we sought to increase engagement and networking via Twitter, with 52 followers. Given these findings, we have incorporated networking activities into upcoming work group meetings about research priorities and continue to share resources and build engagement with the conference attendees through email and Twitter.

Our future activities include three one-hour presentations that will take place virtually, meetings to facilitate workgroup discussions about Health Equity and Implementation and Evaluation, a six-month evaluation of the utility of the conference, dissemination of conference information, and planning for future conference meetings. Future efforts will also be designed to help increase participation in evaluation to improve the response rate and representativeness evaluation responses.

The *Transdisciplinary Conference for Future Leaders in Precision Public Health* was a first-of-its kind conference designed to provide a space for individuals who are invested in precision public health research to convene. We successfully brought together early career researchers working in the field of precision health to learn from established experts, network, and identify opportunities for the future of the field. Our long-term goal is to continue this conference on an annual basis to provide a unique space for the growing field of precision public health.

## Data Availability

Data are available upon request to corresponding author.
